# An Endocrine-Disrupting Chemical, Bisphenol A Diglycidyl Ether (BADGE), Accelerates Neuritogenesis and Outgrowth of Cortical Neurons via the G-Protein-Coupled Estrogen Receptor

**DOI:** 10.3390/neurosci6020053

**Published:** 2025-06-06

**Authors:** Ikuko Miyazaki, Chiharu Nishiyama, Takeru Nagoshi, Akane Miyako, Suzuka Ono, Ichika Misawa, Aika Isse, Kana Tomimoto, Kaori Masai, Kazumasa Zensho, Masato Asanuma

**Affiliations:** 1 Department of Medical Neurobiology, Okayama University Graduate School of Medicine, Dentistry and Pharmaceutical Sciences, Okayama 700-8558, Japan; pzsm58zx@s.okayama-u.ac.jp (K.T.); p1744zqb@s.okayama-u.ac.jp (K.M.); phvw5b1a@s.okayama-u.ac.jp (K.Z.); asachan@cc.okayama-u.ac.jp (M.A.); 2 Department of Medical Neurobiology, Okayama University Medical School, Okayama 700-8558, Japan; me430067@s.okayama-u.ac.jp (C.N.); me430061@s.okayama-u.ac.jp (T.N.); pjai8lce@s.okayama-u.ac.jp (A.M.); pjio4r46@s.okayama-u.ac.jp (S.O.); me502096@s.okayama-u.ac.jp (I.M.); me502008@s.okayama-u.ac.jp (A.I.)

**Keywords:** BADGE, neurite outgrowth, estrogen receptor, GPER, Hes1, neurogenin-3

## Abstract

Bisphenol A diglycidyl ether (BADGE) is the main component of epoxy resin and is used for the inner coating of canned foods and plastic food containers. BADGE can easily migrate from containers and result in food contamination; the compound is known as an endocrine-disrupting chemical. We previously reported that maternal exposure to bisphenol A bis (2,3-dihydroxypropyl) ether (BADGE·2H_2_O), which is the most detected BADGE derivative not only in canned foods but also in human specimens, during gestation and lactation, could accelerate neuronal differentiation in the cortex of fetuses and induce anxiety-like behavior in juvenile mice. In this study, we investigated the effects of low-dose BADGE·2H_2_O (1–100 pM) treatment on neurites and the mechanism of neurite outgrowth in cortical neurons. BADGE·2H_2_O exposure significantly increased the number of dendrites and neurite length in cortical neurons; these accelerating effects were inhibited by estrogen receptor (ER) antagonist ICI 182,780 and G-protein-coupled estrogen receptor (GPER) antagonist G15. BADGE·2H_2_O down-regulated *Hes1* expression, which is a transcriptional repressor, and increased levels of neuritogenic factor neurogenin-3 (Ngn3) in the cortical neurons; the changes were significantly blocked by G15. These data suggest that direct BADGE·2H_2_O exposure can accelerate neuritogenesis and outgrowth in cortical neurons through down-regulation of Hes1 and by increasing Ngn3 levels through ERs, particularly GPER.

## 1. Introduction

Bisphenol A diglycidyl ether (BADGE) is the main component of epoxy resin and is synthesized by reacting bisphenol A (BPA) and epichlorohydrin [1]. BADGE is used for the inner coating of canned foods and plastic containers around the world [1]. It is well known that BADGE percolates from containers during the sterilization process and long-term preservation, with it becoming a food contaminant and leading to human exposure [2,3,4]. BADGE and its hydrolytic and chlorinated derivatives are present in many canned foods [5,6]. Previous studies have shown detection of BAGDE and its derivatives in human specimens such as urine, adipose tissue, serum, and the placenta [7,8,9,10]; in particular, placental transfer of BADGE and its derivatives can lead to prenatal and fetal exposure [10]. BAGDE and its derivatives are documented as endocrine-disrupting chemicals (EDCs); these compounds react with hormone receptors, such as androgenic and estrogenic receptors, and affect hormone action [11,12,13]. Among them, bisphenol A bis (2,3-dihydroxypropyl) ether (BADGE·2H_2_O), a stable hydrated product of BADGE, is detected as a major compound in human blood, adipose, and urine [7,8,14,15] and has estrogenic activity greater than that of BPA [11].

The brain is a target of EDCs, which induce developmental disruption [16]. Exposure to EDCs during the fetal and/or newborn period via placental transport or lactation induced early brain development with neurogenesis, neuronal differentiation, and migration [17,18,19]. We previously examined the effects of BADGE·2H_2_O exposure in dams during gestation and lactation on cortical development and anxiety-related behavior in their offspring [20]. Maternal BADGE·2H_2_O exposure induced the acceleration of neuronal differentiation in fetuses and induced anxiety-like behavior in juvenile mice [20]. Furthermore, we reported that direct BADGE·2H_2_O exposure promoted neurite outgrowth in the primary cultured cortical neurons [20]. However, the mechanism of acceleration of neuronal differentiation induced by BADGE·2H_2_O exposure is still unclear.

The purpose of this study is to elucidate the mechanism of BADGE·2H_2_O-induced neurite outgrowth in cortical neurons. It is well known that BPA mainly binds to estrogen receptors (ERs) such as ERα and ERβ [21]. It is reported that ERβ plays an important role in neural differentiation [22]. Furthermore, the G-protein-coupled estrogen receptor (GPER) has received attention as a potential target of EDCs [23]. Based on these findings, we focused on ERs, classical ERs, and GPER in the acceleration of neurite outgrowth induced by BADGE·2H_2_O exposure. Notch signaling is involved in the control of neurite extension and remodeling in neurodevelopment [24]. Previous studies have indicated that estradiol down-regulated Notch signaling via GPER, which decreased the expression of transcriptional repressor *Hes1*, resulting in increased levels of neuritogenic factor neurogenin-3 (Ngn3), and promoted neuritogenesis [24,25]. Therefore, in order to clarify the signal cascade downstream of GPER, we examined changes in *Hes1* and *Ngn3* mRNA expression and Ngn3 protein levels in cortical neurons after treatment with BADGE·2H_2_O and G15.

## 2. Materials and Methods

### 2.1. Animals

Pregnant Sprague Dawley (SD) rats were purchased from Charles River Japan Inc. (Yokohama, Japan). All of the experimental procedures were conducted according to the NIH Guide for the Care and Use of Experimental Animals and the Policy on the Care and Use of Laboratory Animals, Okayama University, and were approved by the Animal Care and Use Committee, Okayama University (approval reference numbers: OKU-2020006 and OKU-2023162).

### 2.2. Primary Neuronal Culture

Primary cultured neurons were prepared from the cortex of SD rat embryos at 15 days of gestation using a method described previously [20]. To prepare the cortical neuronal cultures, the cortex was dissected and cut into small pieces with scissors. The tissue was incubated in 0.125% trypsin-EDTA at 37 °C for 15 min and then centrifuged (1500× *g*, 3 min). The resulting cell pellet was treated with 0.004% DNase I and 0.003% trypsin inhibitor at 37 °C for 7 min and then centrifuged (1500× *g*, 3 min). The resulting cell pellet was gently resuspended in growth medium: DMEM with high glucose (Invitrogen, San Diego, CA, USA) containing 10% fetal bovine serum (FBS), 4 mM L-glutamine, and 60 mg/L kanamycin sulfate. The cells were plated at a density of 1 × 10^5^ cells/cm^2^ in the poly-D-lysine-coated 4-chamber culture slides (Falcon, Corning, NY, USA) for immunocytochemistry or 6-well plates (Falcon) for quantitative RT-PCR or Western blot analysis. Within 24 h of initial cultivation, the medium was replaced with a fresh growth medium supplemented with 2 µM cytosine-β-D-arabinofuranoside (Ara-C) to inhibit the proliferation of glial cells. Cell cultures were maintained at 37 °C in a 5–95% CO_2_–air gas mixture.

### 2.3. Cell Treatments

BADGE·2H_2_O (Sigma-Aldrich, St. Louis, MO, USA) was freshly prepared in dimethylsulfoxide (DMSO) for each experiment and then diluted to final concentrations in the growth medium (final concentration of DMSO: 0.1% (*v/v*)). The day after initial cultivation, cortical cell cultures were treated with 1–100 pM BADGE·2H_2_O or vehicle (0.1% DMSO; control group) in growth medium. To examine whether BADGE accelerated neurite outgrowth via ERs, neuronal cultures were treated with BADGE·2H_2_O (100 pM) and ICI 182,780 (100 nM, Abcam, Cambridge, UK), classical ERα and ERβ antagonists, or G15 (100 nM, MedChemExpress, Monmouth Junction, NJ, USA), a GPER antagonist, or vehicle (0.1% DMSO) for 6, 24, or 48 h. The cells were cultured in growth medium containing 2 µM Ara-C throughout the culture period.

### 2.4. Immunocytochemistry

Cortical neurons were fixed with 4% paraformaldehyde, blocked with 2.5% normal goat serum for 20 min at room temperature, and then reacted with the following primary antibodies diluted in 10 mM PBS containing 0.1% Triton X-100 (0.1% PBST) for 18 h at 4 °C: mouse anti-microtubule-associated protein 2 (MAP2) (1:5000, M4403, Sigma-Aldrich); rabbit anti-β-tubulin III (1:200, ab52623, Abcam); or mouse anti-pan axonal neurofilament (SMI312) (1:250, 837904, BioLegend, San Diego, CA, USA). After washing in 10 mM PBS (3 × 10 min), the cells were incubated with Alexa Fluor 594-conjugated goat anti-mouse IgG or Alexa Fluor 488-conjugated goat anti-rabbit IgG (1:500, Invitrogen) for 1.5 h at room temperature. The cells were counterstained with Hoechst 33,342 nuclear stain (10 µg/mL) and mounted with Fluorescence Mounting Medium (DakoCytomation, Glostrup, Denmark). All slides were analyzed under a microscope (Olympus BX53, Tokyo, Japan) using the cellSens Standard 1.16 software imaging system (Olympus).

**Figure 1:**
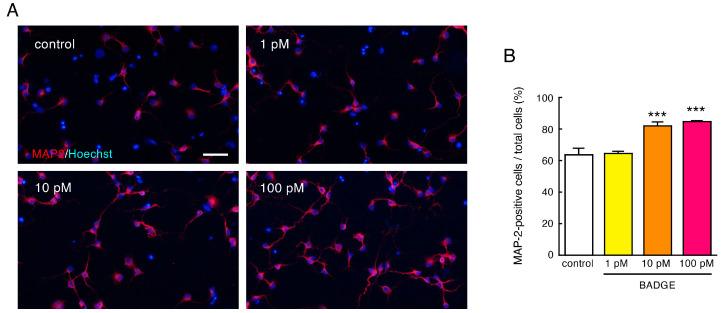

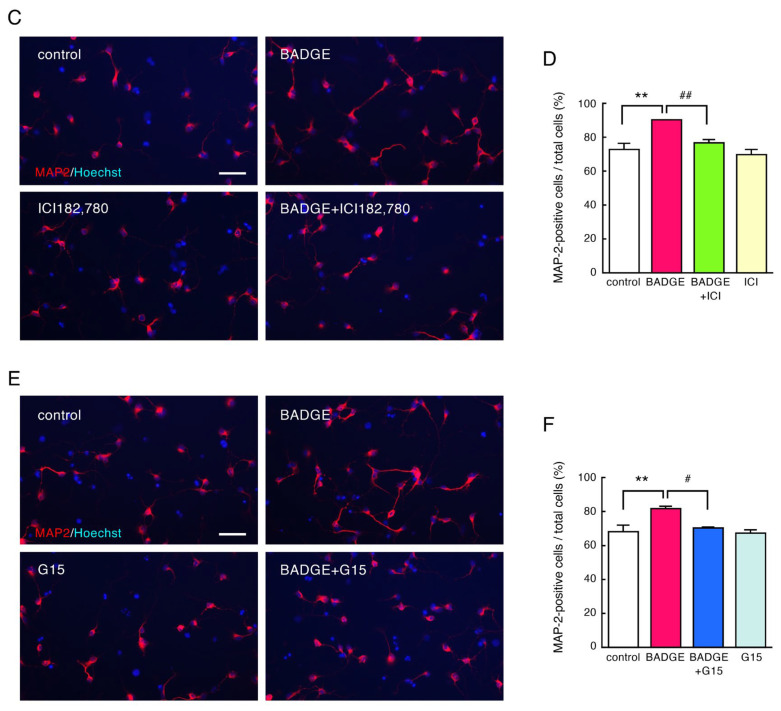
BADGE accelerates neuronal differentiation to mature neurons via ERs. (**A**,**B**) Cortical neuronal cells were treated with BADGE·2H_2_O (1–100 pM) for 48 h. (**A**) MAP2 (red) immunostaining of cortical neuronal cells. Cells were nuclear stained with Hoechst 33,342. Scale bar: 50 µm. (**B**) Quantitation of the images shown in (**A**). Data are the mean ± SEM (*n* = 3 independent cell culture preparations) expressed as percentages of MAP2-immunopositive cells to total cell number. *** *p* < 0.001 vs. control group. (**C**–**F**) ER antagonists, ICI 182,780 (100 nM) (**C**,**D**) and G15 (100 nM) (**E**,**F**), inhibit neuronal differentiation induced by BADGE·2H_2_O (100 pM) exposure. Data are the mean ± SEM (*n* = 3 independent cell culture preparations). ** *p* < 0.01 and *** *p* < 0.001 vs. the control group. ^#^ *p* < 0.05 and ^##^ *p* < 0.01 vs. the BADGE·2H_2_O-treated group.

**Figure 2:**
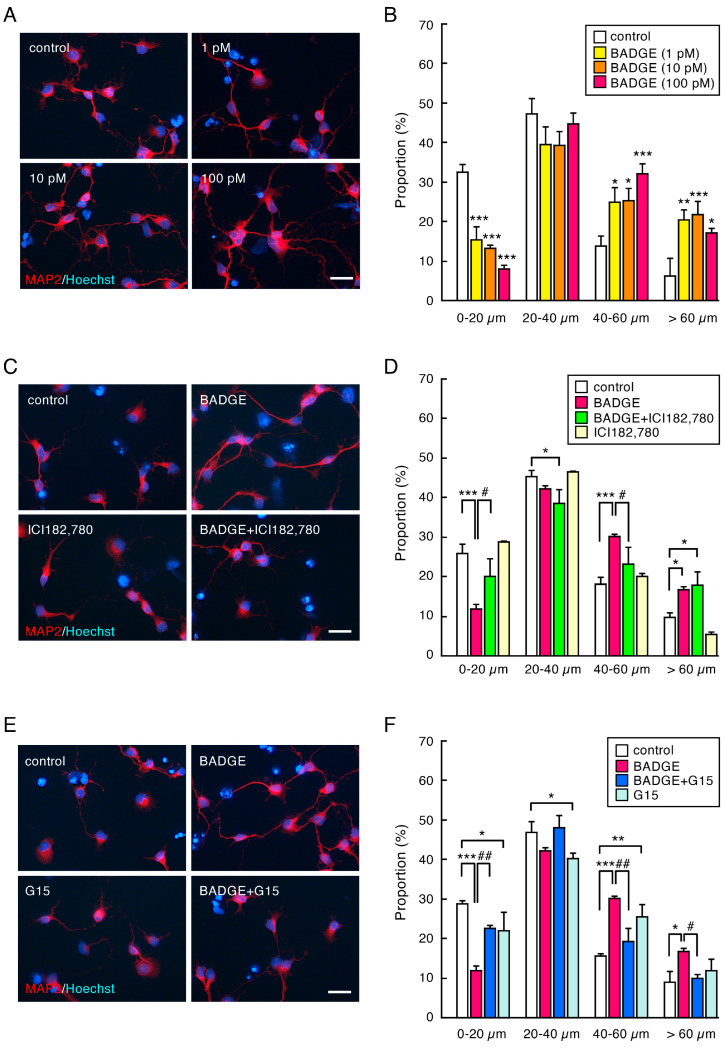
BADGE accelerates neurite outgrowth via ERs. (**A**,**B**) Cortical neuronal cells were treated with BADGE·2H_2_O (1–100 pM) for 48 h. (**A**) MAP2 (red) immunostaining at high magnification. Scale bar: 25 µm. (**B**) Proportion sorted by neurite length. Data are the mean ± SEM (*n* = 3 independent cell culture preparations). * *p* < 0.05, ** *p* < 0.01, and *** *p* < 0.001 vs. the control group. (**C**–**F**) ER antagonists, ICI 182,780 (100 nM) (**C**,**D**) and G15 (100 nM) (**E**,**F**), inhibit neurite outgrowth by BADGE·2H_2_O (100 pM) exposure. Data are the mean ± SEM (*n* = 3 independent cell culture preparations). * *p* < 0.05, ** *p* < 0.01, and *** *p* < 0.001 vs. the control group. ^#^ *p* < 0.05 and ^##^ *p* < 0.01 vs. the BADGE·2H_2_O-treated group.

**Figure 3:**
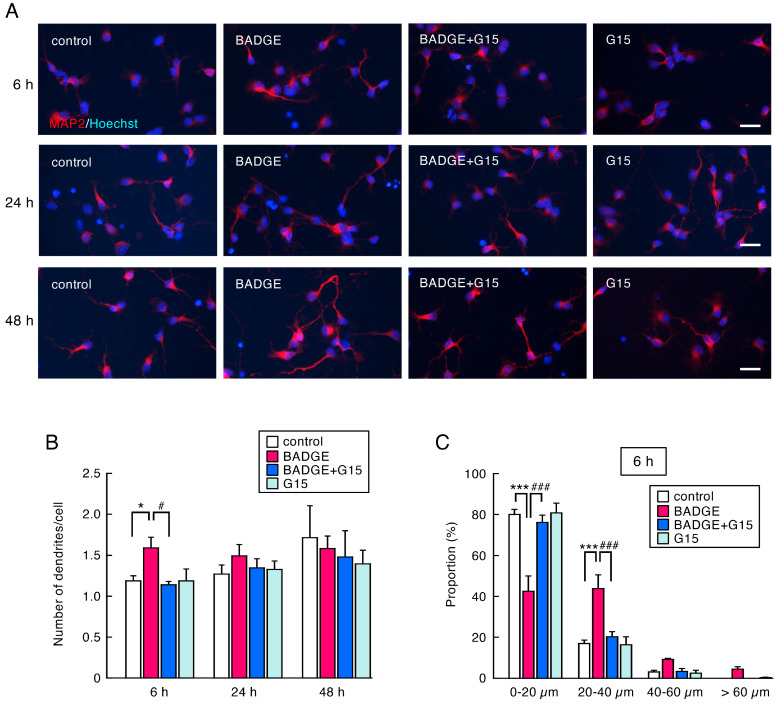

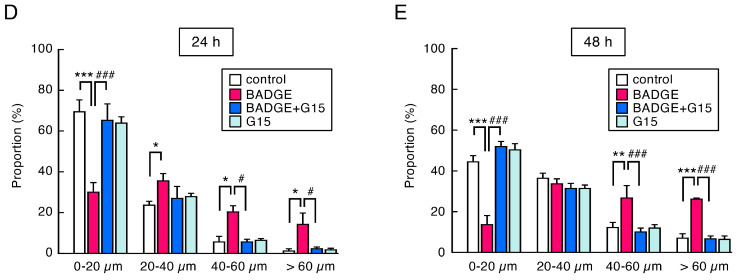
Time-course change in the number of dendrites and neurite length of cortical neuronal cells after treatment with BADGE·2H_2_O and G15. (**A**) MAP2 (red) immunostaining of cortical neuronal cells treated using BADGE·2H_2_O (100 pM) with/without G15 (100 nM) for 6 h, 24 h, and 48 h. Scale bar: 25 µm. (**B**) Number of dendrites per cell. Data are the mean ± SEM (*n* = 3 independent cell culture preparations). * *p* < 0.05 vs. the control group. ^#^ *p* < 0.05 vs. the BADGE·2H_2_O-treated group. (**C**–**E**) Change in proportion sorted by neurite length after treatment with BADGE·2H_2_O (100 pM) and G15 (100 nM) for 6 h (**C**), 24 h (**D**), or 48 h (**E**). Data are the mean ± SEM (*n* = 3 independent cell culture preparations). * *p* < 0.05, ** *p* < 0.01, and *** *p* < 0.001 vs. the control group. ^#^ *p* < 0.05 and ^###^ *p* < 0.001 vs. the BADGE·2H_2_O-treated group.

**Figure 4:**
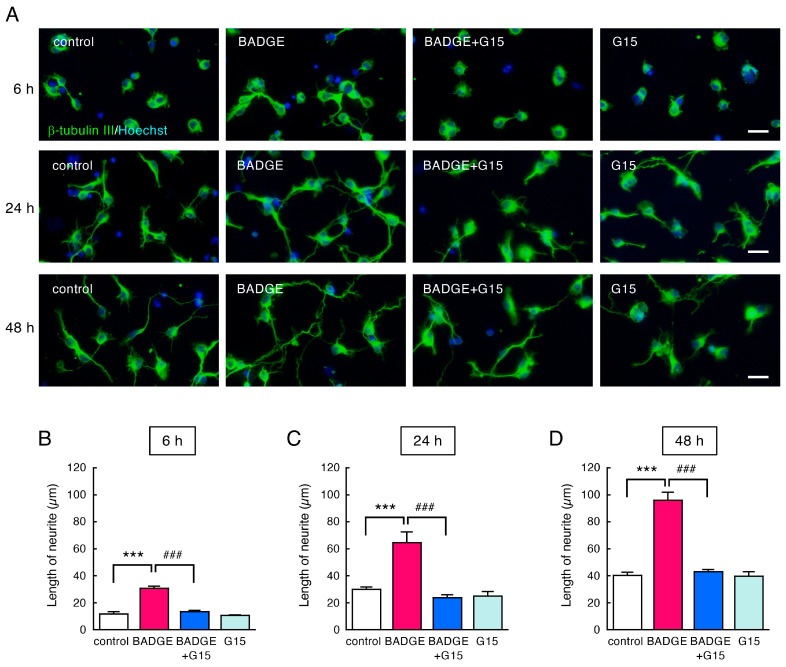
Time-course change in β-tubulin III-positive neurites after treatment with BADGE·2H_2_O and G15. (**A**) β-tubulin III (green) immunostaining of cortical neuronal cells treated using BADGE·2H_2_O (100 pM) with/without G15 (100 nM) for 6 h, 24 h, and 48 h. Scale bar: 25 µm. (**B**–**D**) Change in length of neurites after treatment with BADGE·2H_2_O (100 pM) and G15 (100 nM) for 6 h (**B**), 24 h (**C**), or 48 h (**D**). Data are the mean ± SEM (*n* = 4 independent cell culture preparations). *** *p* < 0.001 vs. the control group. ^###^ *p* < 0.001 vs. the BADGE·2H_2_O-treated group.

**Figure 5:**
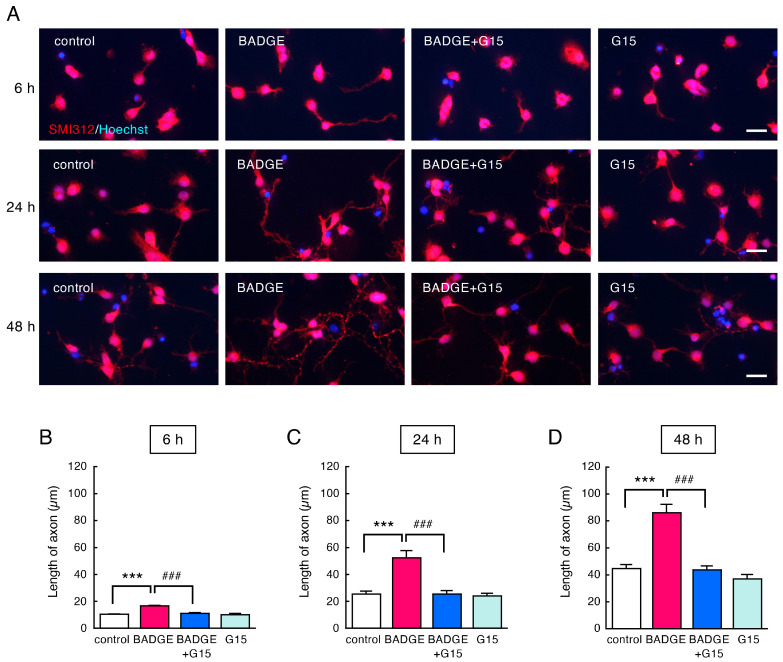
Time-course change in SMI312-positive axons after treatment with BADGE·2H_2_O and G15. (**A**) SMI312 (red) immunostaining of cortical neuronal cells treated using BADGE·2H_2_O (100 pM) with/without G15 (100 nM) for 6 h, 24 h, and 48 h. Scale bar: 25 µm. (**B**–**D**) Change in the length of axons after treatment with BADGE·2H_2_O (100 pM) and G15 (100 nM) for 6 h (**B**), 24 h (**C**), or 48 h (**D**). Data are the mean ± SEM (*n* = 4 independent cell culture preparations). *** *p* < 0.001 vs. the control group. ^###^ *p* < 0.001 vs. the BADGE·2H_2_O-treated group.

The number of MAP2-immunopositive cells in neuronal cultures (Figure 1) was counted under ×200 magnification and expressed as a percentage of MAP2-positive cells to total cell number. The neurite length of MAP2-, β-tubulin III- or SMI312-positive neurons (Figure 2, Figure 3, Figure 4 and Figure 5) was measured under ×400 magnification using a cellSens Standard 1.16 software imaging system. MAP2-positive neurites were sorted by length and expressed as a proportion. The length of β-tubulin III- or SMI312-positive neurites was measured for 15–20 neurons in a captured area, and the average was calculated. The data were collected from 3–4 independent cell culture preparations.

### 2.5. RNA Extraction and Quantitative RT–PCR

Cortical neuronal cells were treated with BADGE·2H_2_O (100 pM) and G15 (100 nM) for 15 h. After treatment, total RNA was extracted from the cells using TRIzol reagent (Invitrogen) and treated with DNase I (Sigma-Aldrich) to remove genomic DNA.

The quantitative RT–PCR was performed with 100 pg of total RNA for *Hes1* or *GAPDH* or 100 ng for *Ngn3* using the One Step TB Green PrimeScript PLUS RT–PCR Kit (Takara Bio Inc., Shiga, Japan). The primers were designed using Primer BLAST (provided by NCBI, NIH) and purchased from Sigma-Aldrich. The primer sequences are as follows: *Hes1* (forward: CAACACGACACCGGACAAAC; reverse: GGAATGCCGGGAGCTATCTT), *Ngn3* (forward: GCAGAGCAGATAAAGCGTGC; reverse: TCGCCTGGAGTAAATTGCGT), *GAPDH* (forward: AGGTCGGTGTGAACGGATTT; reverse: TGGGTTTCCCGTTGATGACC). Quantitative RT–PCR was run using QuantStudio3 (Applied Biosystems, Waltham, MA, USA) and thermocycling program as follows: reverse transcription at 42 °C for 5 min, inactivation of the RT enzyme at 95 °C for 10 s, and 40 cycles (*Hes1* and *GAPDH*) or 45 cycles (*Ngn3* and *GAPDH*) of annealing and extension at 95 °C for 5 s and 60 °C for 30 s. The amplification specificity was confirmed through the analysis of melting curves. The expression levels of *Hes1* and *Ngn3* were normalized to the expression of *GAPDH* and calculated based on the comparative C_t_ method (2^−ΔΔCt^).

### 2.6. Western Blot Analysis

Cortical neuronal cells were treated with BADGE·2H_2_O (100 pM) and G15 (100 nM) for 48 h. After treatment, cytosolic lysates from the cells were extracted using NE-PER™ Nuclear and Cytoplasmic Extraction Reagents (Thermo Fisher Scientific Inc., Rockford, IL, USA). Cultured neuronal cells were lysed through incubation in ice-cold Cytoplasmic Extraction Reagent with a protease inhibitor cocktail (Thermo Fisher Scientific Inc.) for 10 min. After centrifugation (15,000× *g*, 5 min at 4 °C), supernatants were collected. Protein concentrations were measured using the Bio-Rad DC protein assay kit (#5000112JA, Bio-Rad, Richmond, CA, USA).

Protein samples were separated on Any kD SDS-polyacrylamide gels (#4569035, Bio-Rad) and electrophoretically transferred to PVDF membranes (Immobilon-P, Millipore, Temecula, CA, USA). Blots were incubated with mouse monoclonal anti-Ngn3 (1:200, sc-374442, Santa Cruz Biotechnology, Santa Cruz, CA, USA) or goat polyclonal anti-β-actin (1:250, sc-1615, Santa Cruz Biotechnology), followed by incubation with the corresponding HRP-conjugated secondary antibody. Chemiluminescent signals were visualized using the ECL Western blotting detection system (GE Healthcare UK, Buckinghamshire, UK). Images were quantified using a FUJIFILM Luminescent Image Analyzer LAS-3000 (FUJIFILM, Tokyo, Japan) and Multi Gauge (v3.0) software.

### 2.7. Statistical Analysis

Data are presented as the mean ± SEM. Statistical analyses were performed via one-way ANOVA followed by *post hoc* Fisher’s least significant difference (LSD) test using KaleidaGraph v5.0 software (HULINKS Inc., Tokyo, Japan). A *p* < 0.05 was considered statistically significant.

## 3. Results

### 3.1. BADGE Accelerates Neuronal Differentiation to Mature Neurons via ERs

First, we examined the effects of BADGE exposure on neuronal differentiation to mature neurons. Primary cultured cells from the cortex of SD rat embryos at 15 days of gestation were treated with BADGE·2H_2_O (1–100 pM) for 48 h. Treatment with BADGE·2H_2_O (10 and 100 pM) significantly increased the proportion of MAP2-positive mature neurons (Figure 1A,B). The neuronal differentiation induced by BADGE·2H_2_O (100 pM) was completely inhibited by ICI 182,780, the ERα and ERβ antagonist, and G15, the GPER antagonist (Figure 1C–F). These results suggest that BADGE accelerates neuronal differentiation to mature neurons via classical ERs and GPER.

### 3.2. BADGE Accelerates Neurite Outgrowth via ERs

To explore the effects of BADGE exposure on neurite outgrowth, the proportion sorted by neurite length was analyzed. BADGE·2H_2_O treatment promoted neurite outgrowth (Figure 2A). The proportion of cells with short neurites (0–20 µm in length) was significantly decreased by BADGE·2H_2_O (1, 10, 100 and pM) for 48 h, whereas the proportion of cells with long neurites (40–60 µm and >60 µm in length) was dramatically increased (Figure 2B). Neurite outgrowth by BADGE·2H_2_O (100 pM) was partially inhibited by ICI 182,780 (40–60 µm in length; Figure 2C,D) or dramatically by G15 (Figure 2E,F); the inhibition effect of G15 was stronger than that of ICI 182,780. These results suggest that BADGE accelerates neurite outgrowth via classical ERs and GPER; it seems as though GPER is more closely involved in BADGE-induced neurite outgrowth.

### 3.3. Time-Course Analysis of the Number and Length of Neurites After BADGE·2H_2_O Treatment

To examine time-course change in the neuritogenesis and neurite outgrowth, the number and length of neurites were measured after BADGE·2H_2_O (100 pM) exposure for 6 h, 24 h, and 48 h. It has been reported that MAP2, β-tubulin III, and pan neurofilament (SMI312) are markers of neurites: MAP2 for dendrites, β-tubulin III for all neurites, and SMI312 for axons [26]. The number of dendrites significantly increased 6 h after BADGE·2H_2_O exposure, and G15 treatment completely inhibited dendritogenesis (Figure 3A,B). The proportion of cells with short neurites (0–20 µm in length) was significantly decreased by BADGE·2H_2_O at any time point (Figure 3C–E). After 6 h BADGE treatment, the proportion of cells with rather long neurites (20–40 µm in length) increased (Figure 3C). The acceleration of neurite outgrowth by BADGE was clearly observed after 24 h and 48 h of treatment; the proportion of cells with very long neurites (40–60 µm and >60 µm in length) increased (Figure 3D,E). All changes were significantly suppressed by G15 (Figure 3C–E).

Immunostaining for β-tubulin III visualized more neurites, including longer neurites, than MAP2. The β-tubulin III-positive neurites were highly elongated 24 h and 48 h after BADGE·2H_2_O exposure (Figure 4A). BADGE·2H_2_O increased the length of neurites at any time point, and G15 completely inhibited neurite elongation (Figure 4B–D).

The SMI312-positive axons were also elongated after BADGE·2H_2_O treatment at any time point, and G15 completely inhibited axonal elongation (Figure 5).

### 3.4. BADGE Down-Regulates Hes1 Expression and Increases Ngn3 Levels via GPER

**Figure 6:**
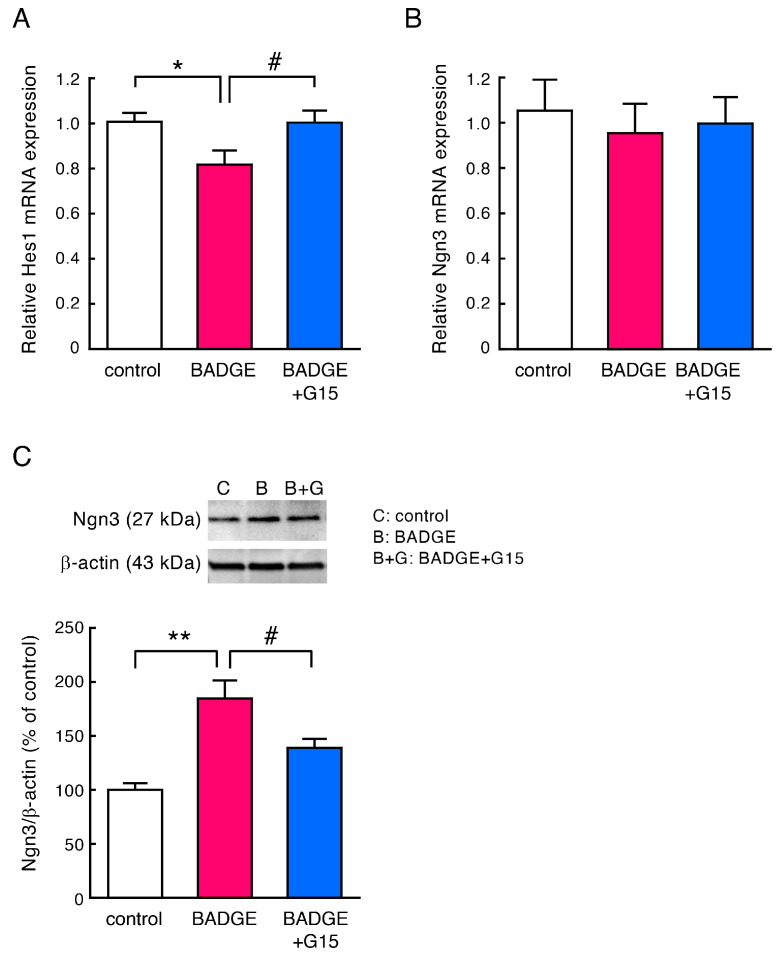
Change in Hes1 and Ngn3 levels after treatment with BADGE·2H_2_O and G15. (**A**,**B**) Quantitative RT-PCR analysis of *Hes1* (**A**) or *Ngn3* (**B**) mRNA expression in cortical neuronal cells after treatment with BADGE·2H_2_O (100 pM) and G15 (100 nM) for 15 h. Data are the mean ± SEM (*n* = 6–9 independent cell culture preparations). (**C**) Western blot analysis of Ngn3 using the cytosolic fraction of cortical neuronal cells after treatment with BADGE·2H_2_O (100 pM) and G15 (100 nM) for 48 h. Top, representative blots; β-actin is used as a loading control. Full Western blot image is provided as Figure S1. Bottom, quantitation of Ngn3. Each value represents the density of the specific protein signal relative to β-actin and is presented as a percentage of the control group. Data are the mean ± SEM (*n* = 3–4 independent cell culture preparations). * *p* < 0.05 and ** *p* < 0.01 vs. the control group. ^#^ *p* < 0.05 vs. the BADGE·2H_2_O-treated group.

Notch signaling is involved in the control of neurite extension and remodeling in neurodevelopment [24]. BPA affects neural differentiation by disrupting Notch signaling [27]. Activation of Notch signaling induces a transcriptional mediator, Hes1, that represses a neuritogenic factor, Ngn3 [28]. It has been reported that estradiol inhibits the Notch pathway via GPER, resulting in down-regulation of *Hes1* mRNA expression and an increase in *Ngn3* mRNA expression, followed by neuritogenesis in hippocampal neurons [24,25]. Therefore, in order to clarify the signal cascade downstream of GPER, we examined changes in *Hes1* and *Ngn3* mRNA expression and Ngn3 protein level in cortical neurons after treatment with BADGE·2H_2_O and G15. BADGE·2H_2_O (100 pM, 15 h) treatment significantly decreased *Hes1* mRNA expression, which was completely inhibited by G15 (Figure 6A); however, there was no change in *Ngn3* mRNA expression (Figure 6B). Ngn3 protein level significantly increased after BADGE·2H_2_O (100 pM) treatment for 48 h, which was significantly inhibited by G15 (Figure 6C).

## 4. Discussion

The present study demonstrates that direct BADGE·2H_2_O exposure at low concentrations can accelerate neuritogenesis and outgrowth in cortical neurons via ER, especially GPER, and that suppression of Hes1 expression and increased Ngn3 levels may be involved in the downstream cascade of GPER.

BADGE is one of the most widely used epoxy resins for coating food and beverage cans and plastic containers. BADGE can be easily transformed to its hydrolysis or chlorinated products in aqueous and acidic foodstuffs during storage; among them, BADGE·2H_2_O is detected as a major compound [5,15]. Wang et al. reported widespread distribution of BADGE and its derivatives in human specimens [7,8,15]. BADGE·2H_2_O was detected in all human urine samples. The detection frequency of BADGE·2H_2_O (60%) in adipose tissue was higher than that of BADGE (25%) [8]. They also reported that BADGE was not detected in plasma samples. In contrast, BADGE·2H_2_O was found in plasma samples with a detection frequency of 70%; the median concentration of BADGE·2H_2_O in plasma samples was 7.15 ng/mL [8]. The concentration is approximately equivalent to 19 nM. In the present study, we treated embryonic cortical neurons with BADGE·2H_2_O at 1–100 pM. Compared to the concentration of BADGE·2H_2_O in human plasma, the doses in our experiments seem extremely low.

In our previous study, we reported that direct BADGE·2H_2_O exposure promoted neurite outgrowth in cultured cortical neurons [20]. In this study, we examined the mechanism of BADGE·2H_2_O-induced acceleration of neurite outgrowth. BADGE·2H_2_O has estrogenic activity greater than that of BPA [11]. Estradiol plays a critical role during early brain development [29,30]; estradiol promotes neuritogenesis, axogenesis, and synaptogenesis [24,31]. The developing brain expresses high levels of estrogen receptors [30]. In particular, it is reported that ERβ is more highly expressed than ERα in the cerebral cortex of neonatal mice [32]; moreover, it is reported that ERβ plays an important role in the neural differentiation of mouse embryonic stem cells [22]. In addition to the classical ERs, ERα and ERβ, GPER is recognized as a critical mediator of rapid signaling in response to estrogen [33,34]. GPER is also highly expressed in the pyramidal cells of the cerebral cortex [35]. Ruiz-Palmero et al. reported that GPER mediated the neuritogenesis induced by 17β-estradiol in hippocampal neurons [36]. Furthermore, GPER has received attention as a potential target of EDCs [23]. Based on these findings, we focused on ERs, especially GPER, as a target of BADGE and examined the involvement of these receptors in the acceleration of neuritogenesis and outgrowth in cortical neurons after direct BADGE·2H_2_O exposure. The acceleration of neuronal differentiation to mature neurons and neurite outgrowth by BADGE·2H_2_O exposure was inhibited by ICI 182,780, the antagonist of ERα and ERβ, and G15, a GPER antagonist; G15 had a stronger inhibitory effect on neurite outgrowth than ICI 182,780. These results suggest that BADGE-induced neuronal differentiation and neurite outgrowth could be mediated via classical ERs, probably ERβ, and GPER. Actually, GPER cross-talks with nuclear ERs; GPER could influence nuclear ER signaling [23,37]. In addition, direct interaction between GPER and ERs is also documented [37]. Therefore, it is unclear whether BADGE·2H_2_O targets only GPER or acts on both ERβ and GPER.

**Figure 7:**
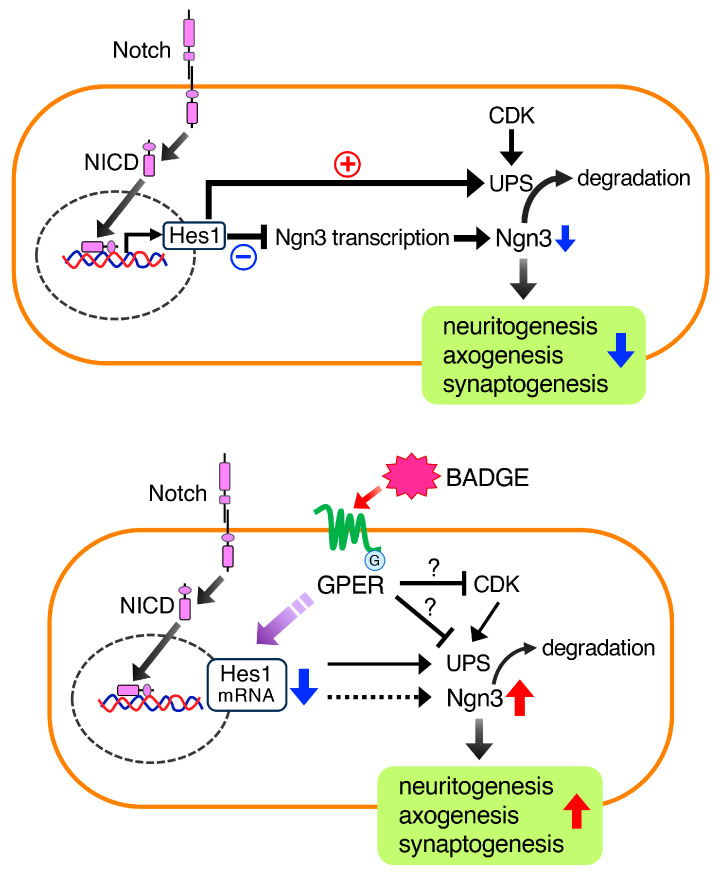
Schematic illustration of the mechanism of BADGE-induced acceleration of neuritogenesis and outgrowth. After activation of Notch signaling, the intracellular domain (NICD) is translocated to the nucleus, enhancing the transcription of Hes1, which inhibits Ngn3 transcription and simultaneously promotes protein degradation by the UPS. Accordingly, Ngn3 is down-regulated, followed by inhibition of neuritogenesis, axogenesis, and synaptogenesis. BADGE down-regulates Hes1 expression and increases Ngn3 levels via GPER. The increase in Ngn3 protein level by BADGE may be due to decreased proteolysis by the UPS caused by down-regulation of Hes1 or due to direct inhibition of the UPS or CDK by BADGE.

Notch signaling is involved in the control of neurite extension and remodeling in neurodevelopment [24]. Intracellularly cleaved and transcriptionally active domain of Notch (NICD) translocates to the nucleus and induces a transcriptional factor, Hes1, which represses Ngn3 [28] (Figure 7). It is well known that Ngn3 promotes neuritogenesis [24,31,38]. A previous study showed that estradiol reduced the levels of NICD in hippocampal slice cultures [39], suggesting that estradiol can down-regulate Notch signaling. In addition, estradiol decreased *Hes1* expression and increased *Ngn3* expression, resulting in neuritogenesis in hippocampal neurons [24,25]. In contrast, G1, a GPER agonist, fully reproduced the effects of estradiol on *Ngn3* expression and neuritogenesis [24]. These findings suggest that the neuritogenic action of estradiol is mediated by GPER. It is also reported that BPA affects neural differentiation by disrupting Notch signaling, leading to neurodevelopmental abnormalities [27]. Based on these findings, we focused on Notch signaling as the signal cascade downstream of GPER, and examined whether BADGE·2H_2_O can down-regulate Notch signaling, reduce Hes1, and consequently up-regulate Ngn3. Because the level of NICD in the cortical neurons was very low in our study, we could not detect NICD in the nuclei of neurons. Next, we examined changes in *Hes1* and *Ngn3* mRNA expression via quantitative RT-PCR. BADGE·2H_2_O treatment significantly decreased *Hes1* mRNA expression, which was completely inhibited by GPER antagonist G15; however, there was no change in *Ngn3* mRNA expression. BADGE·2H_2_O exposure significantly increased Ngn3 protein levels in cortical neurons, which were moderately inhibited by G15. Levels of Ngn3 are regulated not only by transcriptional factor Hes1 but also via proteolysis by the ubiquitin–proteasome system (UPS) or cyclin-dependent kinase (CDK) [40]. In addition, Hes1 suppresses Ngn3 levels not only through direct transcriptional repression but also by promoting protein degradation by the UPS [41]. Therefore, the increase in Ngn3 protein level induced by BADGE·2H_2_O may not be due to transcriptional activation caused by down-regulation of Hes1, but rather due to decreased proteolysis by the UPS regulated by Hes1, resulting in protein accumulation (Figure 7). Similar reports have shown that GPER agonist G1 increased the protein level of the glycolytic enzyme activator 6-phosphofructo-2-kinase/fructose-2,6-biphosphatase 3 (PFKFB3) by inhibiting the UPS without affecting its mRNA expression [42]. Furthermore, it has been reported that estrogen decreases the proliferation of neural stem cells through inhibition of CDK caused by up-regulation of the CDK inhibitor p21 [43]. Considering these findings, it is also possible that BADGE·2H_2_O directly inhibits the UPS or CDK (Figure 7). Further investigation is required to determine whether BADGE·2H_2_O inhibits the nuclear translocation of NICD and the UPS or CDK to suppress the proteolysis of Ngn3.

The brain is a target of EDCs, which disrupt neural development [16]. Exposure to EDCs during fetal and/or neonatal periods via placental transport or lactation induces early brain development with neurogenesis, neuronal differentiation, and migration [17,18,19]. Early brain development, accompanied by an increase in cell proliferation and acceleration of neuronal differentiation, is related to autism spectrum disorder [44]. Therefore, BADGE exposure during fetal development or infancy could be a serious problem. Zhang et al. reported placental transfer of BADGE and its derivatives [10]. They determined BADGE and derivatives in maternal and umbilical cord serum samples and demonstrated high accumulation of BADGE·2H_2_O in cord serum [10]. In addition, Yang et al. detected BADGE and derivatives in human breast milk using ultra-high-performance liquid chromatography–tandem mass spectrometry [45]. BADGE is used in medical devices, such as infusion sets, injection syringes, and catheters. It has recently been reported that BADGE·2H_2_O is detected in the serum of all infants with a history of neonatal intensive care unit hospitalization [46]. In our previous report, we demonstrated that maternal BADGE·2H_2_O exposure during the gestation and lactation periods induced acceleration of neuronal differentiation in fetuses [20]. In the present study, we demonstrated that direct and extremely low-dose BADGE·2H_2_O (1–100 pM) exposure accelerated neurite outgrowth in embryonic cortical neurons. Taken together, this study underscores the risk that maternal BADGE exposure, especially during pregnancy and lactation, could cause abnormal early brain development in the fetus. To avoid carcinogenicity and genotoxicity, the tolerable daily intake (TDI) of BADGE is established at less than 0.15 mg/kg/day by the European Food Safety Authority (EFSA) [47]. The present findings can contribute to the review of the TDI of BADGE based on not only carcinogenicity and teratogenicity but also adverse neurodevelopmental effects in the central nervous system.

## 5. Conclusions

We have reported that BADGE·2H_2_O, which is a highly detected EDC in human specimens, promotes neuritogenesis and outgrowth mainly via GPER and that the cascade following GPER involves decreased *Hes1* expression and increased Ngn3 levels. This study demonstrates the neurological effects of very low doses of BADGE·2H_2_O and raises the issue of central nervous system exposure to EDC.

## Data Availability

The raw data supporting the conclusions of this article will be made available by the authors upon request.

## Supplementary Materials

The following supporting information can be downloaded at: https://www.mdpi.com/article/10.3390/neurosci6020053/s1. Figure S1: Full Western blot image.
