# Supplementary figures and images for: An Endocrine-Disrupting Chemical, Bisphenol A Diglycidyl Ether (BADGE), Accelerates Neuritogenesis and Outgrowth of Cortical Neurons via the G-Protein-Coupled Estrogen Receptor
Source: NeuroSci. 2025 Jun 6;6(2):53. doi: 10.3390/neurosci6020053 (PMC12196484; doi:10.3390/neurosci6020053)

Full-length blots of Western blotting (Figure 6C)

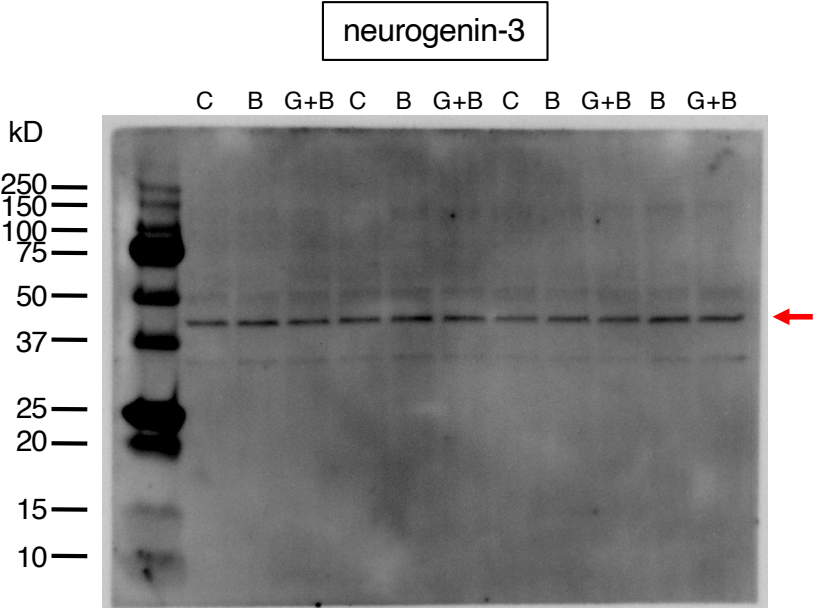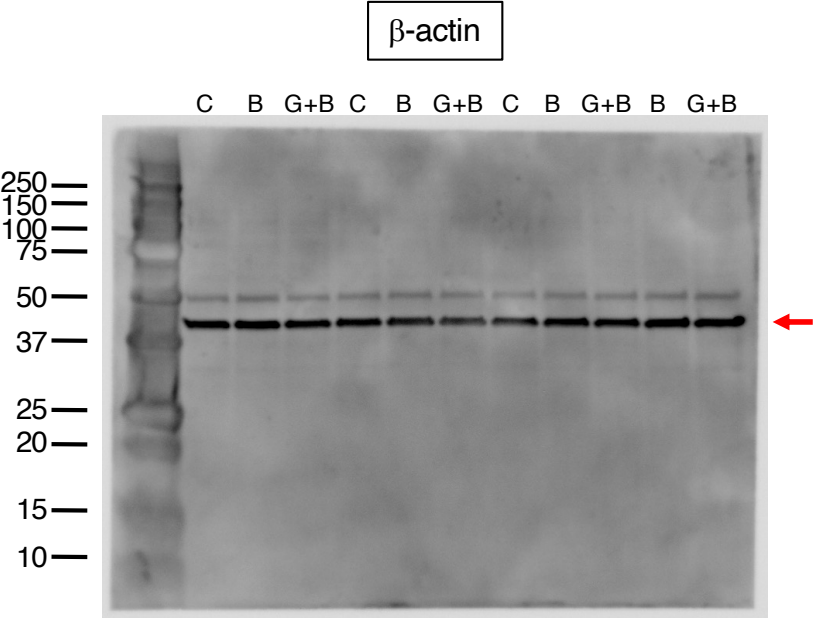

C: control  
B: BADGE  
G+B: G15+BADGE

Supplement: Supplementary file 1 [file neurosci-06-00053-s001.zip › neurosci-3416473-supplementary.pdf]
